# Emotional Expression and Mental Health Support in BTS Fandom Communities Using Natural Language Processing on YouTube Comments: Cross-Sectional Study

**DOI:** 10.2196/74397

**Published:** 2026-08-31

**Authors:** Nari Yoo, Aaron H Rodwin, Michael Park, Sangpil Youm, Sou Hyun Jang

**Affiliations:** 1 School of Social Work University of Michigan Ann Arbor, MI United States; 2 Silver School of Social Work New York University New York, NY United States; 3 School of Social Work Rutgers, The State University of New Jersey New Brunswick, NJ United States; 4 Department of Computer & Information Science & Engineering University of Florida Gainesville, FL United States; 5 Department of Sociology Korea University Seoul Republic of Korea

**Keywords:** BTS, emotional expression, K-pop fandom, LIWC, mental health, music-based coping, natural language processing, online peer support, Taylor Swift, YouTube comments

## Abstract

**Background:**

The global rise of K-pop has shaped youth culture and online communities, particularly through BTS, a South Korean boy band with an international fanbase known as ARMY (Adorable Representative MC for Youth). Music fandoms are increasingly engaging with digital platforms such as YouTube not only for entertainment but also as spaces for emotional expression and mutual support. Despite growing interest in the mental health potential of music-based coping strategies, limited research has examined how fandom cultures differentially express emotional needs and supportive interactions online.

**Objective:**

This study investigates specific mental health language patterns and coping mechanisms expressed by BTS fans in online spaces, examining how different linguistic features (including self-referential language and emotional expression patterns) may reflect psychological states and mental health needs. We used YouTube comments from fan-curated “sad” or “depression” playlists of BTS. We further included YouTube comments from equivalent Taylor Swift playlists as a reference group.

**Methods:**

Using natural language processing and Linguistic Inquiry and Word Count 2022 software, we analyzed a total of 13,224 YouTube comments: 11,772 comments on BTS "sad playlist" videos and 1452 comments on Taylor Swift equivalents. Statistical comparisons were conducted to evaluate differences in comment length, word count, pronoun use, and emotional valence. Representative comments were examined to contextualize the emotion classification results.

**Results:**

BTS original comments were significantly longer (mean 253.38, SD 703.65 characters) and had higher word counts (mean 38.93, SD 88.54 words) than Taylor Swift original comments (length: mean 89.84, SD 330.96 characters; word count: mean 16.08, SD 64.78 words; P<.001). BTS fans used more first-person singular pronouns (mean 10.24%, SD 9.57% vs mean 7.43%, SD 9.41%) and expressed greater sadness (1691/5341, 31.7% vs 75/452, 16.6%). In contrast, Taylor Swift fans exhibited higher admiration (86/452, 19% vs 429/5341, 8%). Among reply comments, BTS fans demonstrated more caring (242/1729, 14% vs 7/128, 5.5%), gratitude (294/1729, 17% vs 15/128, 11.7%), and optimism (162/1729, 9.4% vs 6/128, 4.7%). Linguistic analysis also revealed a broader international user base for BTS, including higher proportions of Spanish (719/11,772, 6.11%) and Portuguese (222/11,772, 1.89%) comments. Examination of comment content showed that fans used these spaces to disclose personal struggles, express gratitude for the community, and offer peer support, with many describing the fandom as a safe space for emotional expression they could not access elsewhere.

**Conclusions:**

The findings show that comments on BTS fan playlists included more emotional disclosure and more supportive replies than those on the Taylor Swift comparison, consistent with fan communities operating as informal sites of peer support online. These results suggest implications for culturally responsive, community-based, and digitally mediated mental health interventions among youth and global populations.

## Introduction

### Overview

Mental health conditions are a growing public health concern, with rates of depression, anxiety, and suicidality increasing across populations [1,2]. Suicide rates in the United States have risen by 57% among individuals aged 10-24 years between 2007 and 2018 [3,4], with similar trends observed globally [5]. Despite the availability of treatments, engagement with mental health services remains low, with many individuals experiencing structural, social, and psychological barriers to care [6,7]. This treatment gap has led researchers to examine alternative sources of mental health expression, support, and engagement, particularly in digital spaces.

Social media platforms contain large-scale, user-generated text that can provide insight into how individuals discuss mental health [8], seek support [9], and describe their psychological states [10]. YouTube, as one of the largest video-sharing platforms, hosts discussions around mental health through comments, vlogs, and music engagement [11,12]. Prior research has demonstrated that individuals often disclose psychological distress in these settings, creating opportunities to study mental health–related language and emotional expression at scale [13].

To specifically investigate coping mechanisms related to depressive mood, this study focuses on comments from YouTube playlists explicitly curated by fans and labeled as “sad” or “depression” playlists. This targeted approach allows for an in-depth examination of emotional expression and support-seeking in a context where users likely engage with music for mood regulation [14-16]. As interactions on online platforms, such as YouTube, have been described as a “clinical whitespace,” they provide new avenues for early detection and monitoring, which holds importance for clinical practice [17]. Further, music use has long been associated with emotional regulation, coping, and psychological well-being [18,19]. It can support emotional processing, evoke memories, and provide an outlet for self-expression [20,21].

### Music, Emotions, and Mental Health

Music is widely used as a tool for emotional processing and psychological well-being [18]. It can influence mood regulation, social connection, and personal identity [20]. Studies have found that music-based interventions can improve mental health outcomes, particularly in therapeutic settings [19,22,23]. However, music engagement can also serve maladaptive functions with mixed effects, as some individuals may use music to cope in ways that reinforce distress and intensify rumination [24,25]. A growing body of research, including reports from the World Health Organization [19] and the National Institutes of Health [22], suggests that psychosocial interventions that integrate music are associated with improved engagement in treatment and mental health outcomes [23,26]. This research highlights the multisensory nature of music and its capacity to evoke emotions, imagination, and activate the senses [19,22].

### Music Engagement and Mental Health Expression

Music listening is often an emotionally charged experience, and online platforms provide opportunities for individuals to share their interpretations, reactions, and personal connections to music. Prior studies have examined how song lyrics reference mental health themes and how music can influence help-seeking behaviors [27,28]. The impact of music on mental health extends beyond lyrical content to the ways in which listeners engage with and discuss music online [29,30].

Studies on music-based online communities suggest that fandoms may operate as spaces for social support, shared identity, and mental health discourse [31-33]. While music engagement can provide comfort and validation, it can also reinforce distress through maladaptive listening patterns, including rumination and avoidance [25,34,35]. An increasing body of work has begun to examine the connection between music, fandom, and well-being. For example, Laffan and colleagues [36] revealed that BTS fans’ psychological sense of community was a protective factor against the impacts of cyberbullying. Recent work has also addressed the psychological dimensions of music fandom: Pownall [37] offered reflections on how Taylor Swift’s lyrics relate to fans’ developmental experiences, while Baudinette and Scholes [38] found that K-pop fandom provides affective spaces of security and support for identity exploration. However, less research has examined how fan communities articulate emotional needs related to depressive mood in online spaces such as YouTube comments, and how these expressions manifest linguistically.

While numerous K-pop groups have gained international popularity, BTS (a K-pop boy band) is distinctive for the consistent themes of self-love, personal growth, and mental health awareness [39]. For example, a recent Billboard survey [40] reported that 62% of respondents said K-pop helps relieve stress and enhance a sense of community belonging. This focus aligns closely with the study’s objectives, making BTS’s music and fan interactions especially relevant. BTS’s massive and highly engaged global fanbase, known as ARMY (Adorable Representative MC for Youth), provides a novel data source for examining the intersection of music engagement and mental health across different cultures and demographics. For these reasons, BTS was selected as the focus for this study to gain perspectives on the intersection of music engagement and mental health [41,42].

While the focus of this study is BTS for the reasons discussed above, we also include Taylor Swift as a reference group, as she is the world’s most streamed artist [43], and her music also contains mental health–related content [29,44]. Both fandoms illustrate the reach of parasocial relationships in the digital age [44-46]. However, the BTS ARMY stands out in its global reach and cultural diversity. While Swift’s fandom is predominantly American, presented as Miss Americana [47], BTS has cultivated a truly international following, bridging language and culture [39].

### Mental Health Discourse on YouTube Comments

Social media platforms provide large-scale data sources for analyzing mental health–related language behavior [48] and help-seeking behavior [9]. While prior research has primarily used data from platforms such as Reddit [49] and Twitter [50], YouTube has become a relevant platform for studying mental health discourse, as users engage with content that reflects their emotional states, including music playlists labeled with mental health–related themes [12,51]. Prior studies have documented how YouTube comments can provide a form of peer support, self-expression, or stigma reduction [13].

In contrast to platforms such as Reddit, where discussions are more text-based, YouTube’s comment sections are tied to audiovisual content, influencing the way users articulate emotional experiences and allowing for more contextually grounded emotional expression [11]. Music-based playlists labeled as “sad” or “depression” provide a space where users engage in discussions about their mental health experiences, often in response to the emotional tone of the music. Prior research has suggested that such spaces can operate as informal support systems while also carrying risks of reinforcing distress [51].

### Objective

This study examines how BTS fans express emotional needs and engage in peer support within YouTube comment sections on “sad” or “depression” playlists. Using natural language processing (NLP) techniques, we analyze linguistic patterns and emotional expressions in fan comments collected between 2018 and 2024. We address the following two research questions: (1) What linguistic characteristics and emotional tones characterize BTS fan comments across this period? (2) How do linguistic features relate to different types of emotional expression in these online communities? Taylor Swift fan comments were included as a reference group to contextualize findings.

## Methods

### Dataset

This study uses established NLP techniques to analyze the linguistic and emotional characteristics of YouTube comments within mental health–themed music engagement. YouTube was selected as the data source for this study, given its position as one of the largest platforms for music-related fan engagement, with comment sections that allow extended textual responses suitable for linguistic analysis of emotional expression and peer support [11]. The data consists of YouTube comments collected from the BTS “sad” or “depression” playlists. While the primary conceptual focus of this study is on BTS fandom, we also included Taylor Swift’s “sad” or “depression” playlists as a reference group to enhance interpretability and context for results. The YouTube comments were collected using the YouTube application programming interface via Google. By using the search terms “sad playlist” and “depression playlist” with “BTS” or “Taylor Swift,” we collected the video IDs for 16 videos for the BTS playlist and 14 videos for the Taylor Swift playlist.

The collected comments were categorized into 2 types: original comments and replies. Original comments refer to stand-alone comments made directly on the video. Reply comments, on the other hand, are responses to original comments, representing fan engagement with other fans and their opinions. In the analysis, we included 11,772 comments from 9062 users for BTS and 1452 comments from 1067 users for Taylor Swift. A total of 46 comments (23 BTS and 23 Taylor Swift) consisted of a single character and returned no analyzable text in Linguistic Inquiry and Word Count 2022 software (LIWC), so the linguistic analyses in the Results section are based on 13,178 comments (BTS: n=11,749; Taylor Swift: n=1429), and the models are based on 12,786 comments after listwise deletion of comments with no LIWC dictionary match. The data were collected in May 2024, covering comments published from October 21, 2018, to May 23, 2024, representing the entire range available on the data collection date. This study uses a cross-sectional design, analyzing aggregated patterns across this period rather than examining temporal trends. This approach enables characterization of overall linguistic and emotional patterns within fan communities while maximizing sample size for robust statistical analysis.

We included the comments written in non-English languages using the Helsinki model [52]. The Helsinki-NLP models were selected for their broad language coverage across over 1000 language pairs and demonstrated strong performance in open-domain translation benchmarks [53]. Among publicly available neural machine translation systems, OPUS-MT (Open Parallel Corpus machine translation) models have been widely adopted in multilingual NLP research due to their accessibility and consistent performance across low- and high-resource languages [53]. BTS fans used a diverse range of languages in their comments, with English (n=8963, 76.14%) being the most common, followed by Spanish (n=719, 6.11%), Portuguese (n=222, 1.89%), Somali (n=140, 1.19%), German (n=98, 0.83%), Indonesian (n=94, 0.8%), Korean (n=76, 0.65%), and Russian (n=69, 0.59%). In contrast, Taylor Swift fans are significantly more likely to use English (n=1166, 80.3%), with Spanish (n=42, 2.89%) and German (n=35, 2.41%) being the only other languages representing more than 2% of the comments. Other languages included Dutch (n=15, 1.03%), Norwegian (n=13, 0.9%), Indonesian (n=11, 0.76%), Somali (n=11, 0.76%), Welsh (n=10, 0.69%), Estonian (n=9, 0.62%), Afrikaans (n=7, 0.48%), Danish (n=7, 0.48%), Romanian (n=7, 0.48%), and Portuguese (n=6, 0.41%; *χ*^2^_45_=151.52; *P*<.001).

### Data Analysis

**Figure 1:**
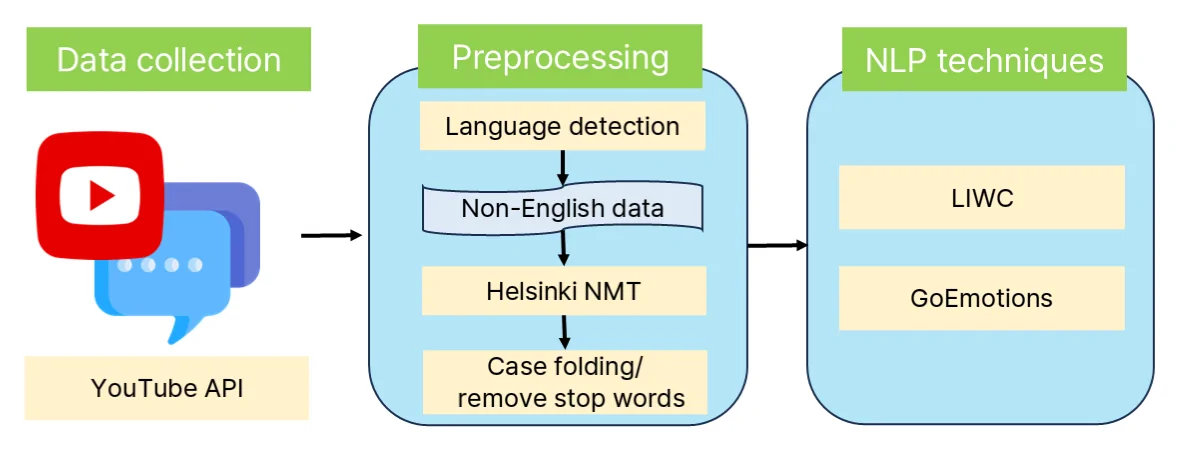
Flowchart of study design. API: application programming interface; LIWC: Linguistic Inquiry and Word Count 2022 software; NLP: natural language processing; NMT: neural machine translation.

A study design is depicted in Figure 1. The research began with applying NLP techniques to the entire dataset of YouTube comments. The data preprocessing stage involved standardizing text by converting to lowercase, removing URLs, special characters, and excessive whitespace. Stop words were removed using the standard English stop-word list from the Natural Language Toolkit library. Tokenization was performed using the *spaCy* library’s default English tokenizer, which segments text based on punctuation and whitespace. For comments translated into English, these preprocessing steps were applied post translation.

Linguistic analysis was conducted using the LIWC with its default English dictionary [54]. We focused on first-person singular pronouns (“I”) as a key linguistic marker because previous research has established strong associations between higher “I” usage and depression, psychological distress, and self-focus during emotional processing [55,56]. Similarly, first-person plural pronouns (“we”) were analyzed to examine collective identity and social connection [57]. Word count and comment length were treated as indicators of emotional expressiveness and disclosure depth, which typically increase when processing difficult emotions or seeking support. Independent sample *t* tests were conducted to compare the differences between BTS’s and Taylor Swift’s comments.

The Bidirectional Encoder Representations from Transformers (BERT)–based GoEmotions model was used to predict 27 types of emotions [58] to analyze the range of emotions experienced by fans as they engaged with the music. The GoEmotions model was selected as one of the most fine-grained emotion classification models available, trained on 58,000 Reddit comments [58]. The model has been widely used in social media emotion analysis due to its granular emotion taxonomy and strong benchmark performance [59], and it was applied to comments that were either originally in English or translated into English [60,61]. We used the pretrained Google BERT model fine-tuned on the GoEmotions Reddit dataset without further fine-tuning on our specific dataset, using default inference parameters.

After inference, chi-square tests were conducted to compare emotions across BTS and Taylor Swift. To contextualize the quantitative emotion classification results, we systematically examined the content of comments within each emotion category. This approach allowed for an in-depth exploration of how fans articulated mental health–related experiences and engaged in peer support. Representative comments from each emotion category were selected to illustrate the range of emotional expressions and support dynamics present in the data.

To analyze how fandom affiliation and linguistic features are associated with emotional expression in comments, we constructed multilevel logistic regression models for the 5 most frequent emotions identified in our initial analysis. We used multilevel modeling with random intercepts for video ID to account for potential clustering effects, as comments within the same video might share similar emotional characteristics. The dependent variable was the binary presence (1) or absence (0) of each emotion as classified by the BERT-based GoEmotions model. Independent variables included fandom (BTS vs Taylor Swift), comment type (reply vs original), number of replies received, comment length, word count, first-person singular pronoun usage (“I”), first-person plural pronoun usage (“we”), and affiliation score from LIWC analysis.

### Ethical Considerations

This study analyzed only publicly available YouTube comments and involved no interaction or intervention with individuals and no access to private identifiable information. It therefore did not meet the regulatory definition of human subjects research under 45 CFR 46.102, and institutional review board review was not sought. Ethical considerations nonetheless remain regarding user consent and privacy. While public data may not legally require informed consent, ethical guidelines emphasize respecting user expectations and minimizing harm. Users may not anticipate their comments being analyzed for research, raising concerns about privacy and autonomy. Following best practices in social media research [62], we did not analyze user IDs, usernames, or other identifiable information, ensuring that individual commenters could not be traced. Further, we still follow common ethical standards, such as removing all personally identifiable information from the comments that we report. No informed consent was obtained and no compensation was provided because the study involved no recruitment of, or interaction with, individuals.

## Results

### Linguistic Analysis

Word frequency analysis showed that the term “ARMY” was mentioned in 1253 (10.64%) BTS-related comments, whereas “Swiftie” was mentioned in 10 (0.69%) Taylor Swift–related comments.

The LIWC analysis revealed significant differences in several linguistic features between comments related to BTS and Taylor Swift (Table 1). For original comments, the average length of comments was significantly longer for BTS (mean 253.38, SD 703.65 characters) compared to Taylor Swift (mean 89.84, SD 330.96 characters), with a *P* value of <.001. The word counts also showed a significant difference, with BTS-related comments averaging 38.93 (SD 88.54) words and Taylor Swift–related comments averaging 16.08 (SD 64.78) words (*P*<.001).

**Table 1:** Comparison of linguistic characteristics across BTS and Taylor Swift comments. Length is in characters; word count is in words; I, we, and affiliation are Linguistic Inquiry and Word Count 2022 software scores expressed as a percentage of total words.

Characteristic		Total	BTS	Taylor Swift	*P* value^a^
Original comments					
	Count, n	9597	8522	1075	N/A^b^
	Number of replies, mean (SD)	0.9 (8.8)	0.9 (9.3)	0.5 (2.3)	.10
	Length, mean (SD)	235.1 (674.2)	253.4 (703.6)	89.8 (331.0)	<.001
	Word count, mean (SD)	36.4 (86.5)	38.9 (88.5)	16.1 (64.8)	<.001
	I, mean (SD)	9.9 (9.6)	10.24 (9.57)	7.4 (9.4)	<.001
	We, mean (SD)	0.5 (2.2)	0.5 (2.2)	0.4 (2.1)	.08
	Affiliation, mean (SD)	1.4 (3.9)	1.4 (3.7)	1.2 (5.2)	.08
Replies					
	Count, n	3581	3227	354	N/A
	Length, mean (SD)	99.1 (179.6)	104.0 (185.8)	54.8 (98.2)	<.001
	Word count, mean (SD)	18.1 (35.2)	19.2 (36.7)	8.7 (11.4)	<.001
	I, mean (SD)	7.9 (11.9)	8.0 (11.9)	6.4 (11.2)	.02
	We, mean (SD)	0.9 (4.3)	1.0 (4.4)	0.4 (2.3)	.02
	Affiliation, mean (SD)	2.3 (6.6)	2.4 (6.8)	1.0 (3.7)	<.001

^a^*P* values are calculated based on the *t* tests for the continuous variables (number of replies, length, word count, I, we, and affiliation).

^b^N/A: not applicable.

The usage of the pronoun “I” was higher in BTS-related comments (mean 10.24%, SD 9.57%) compared to Taylor Swift (mean 7.43, SD 9.41), with a *P* value of <.001. The usage of the pronoun “we” did not show a significant difference (*P*=.08). For reply, as depicted in Table 1, the average length of comments was significantly longer for BTS (mean 103.98, SD 185.75 characters) compared to Taylor Swift (mean 54.77, SD 98.23 characters), with a *P* value of <.001.

The word counts also showed a significant difference, with BTS-related comments averaging 19.17 (SD 36.74) words and Taylor Swift–related comments averaging 8.75 (SD 11.40) words (*P*<.001). The usage of the pronoun “I” was higher in BTS-related comments (mean 8.04, SD 11.92) compared to Taylor Swift (mean 6.43, SD 11.23), with a *P* value of .02. The usage of the pronoun “we” was significantly higher in BTS-related comments (mean 1.00, SD 4.44) compared to Taylor Swift (mean 0.43, SD 2.29), with a *P* value of .02. Affiliation scores were significantly higher for BTS (mean 2.43, SD 6.85) compared to Taylor Swift (mean 0.97, SD 3.69), with a *P* value of <.001.

### Emotion Classification

As depicted in Table 2, the distribution of emotion based on the emotion classification of BTS- and Taylor Swift–related comments was significantly different across both the comments and replies (comments: *χ*^2^_23_=189.0; replies: *χ*^2^_24_=66.3; both *P*<.001).

**Table 2:** Emotion classification across BTS and Taylor Swift (TS) comments and replies. Percentages are calculated among comments (or replies) that the GoEmotions classifier assigned to a nonneutral emotion category: comments: n=5793 (BTS: n=5341; Taylor Swift: n=452); replies: n=1857 (BTS: n=1729; Taylor Swift: n=128).

Emotion	Original comments					Replies			
	Total, n (%)	BTS, n (%)	TS, n (%)	BTS-TS difference (%)	Total, n (%)		BTS, n (%)	TS, n (%)	BTS-TS difference (%)
Admiration	515 (8.9)	429 (8)	86 (19)	–11	170 (9.2)		150 (8.7)	20 (15.6)	–6.9
Amusement	106 (1.8)	90 (1.7)	16 (3.5)	–1.8	54 (2.9)		41 (2.4)	13 (10.2)	–7.8
Anger	55 (0.9)	49 (0.9)	6 (1.3)	–0.4	10 (0.5)		9 (0.5)	1 (0.8)	–0.3
Annoyance	41 (0.7)	39 (0.7)	2 (0.4)	0.3	15 (0.8)		13 (0.8)	2 (1.6)	–0.8
Approval	131 (2.3)	107 (2)	24 (5.3)	–3.3	84 (4.5)		76 (4.4)	8 (6.3)	–1.9
Caring	152 (2.6)	143 (2.7)	9 (2)	0.7	249 (13.4)		242 (14)	7 (5.5)	8.5
Confusion	47 (0.8)	42 (0.8)	5 (1.1)	–0.3	14 (0.8)		12 (0.7)	2 (1.6)	–0.9
Curiosity	31 (0.5)	23 (0.4)	8 (1.8)	–1.4	11 (0.6)		11 (0.6)	0 (0)	0.6
Desire	212 (3.7)	207 (3.9)	5 (1.1)	2.8	45 (2.4)		43 (2.5)	2 (1.6)	0.9
Disappointment	171 (3)	167 (3.1)	4 (0.9)	2.2	21 (1.1)		17 (1)	4 (3.1)	–2.1
Disapproval	33 (0.6)	28 (0.5)	5 (1.1)	–0.6	12 (0.6)		9 (0.5)	3 (2.3)	–1.8
Disgust	10 (0.2)	10 (0.2)	0 (0)	0.2	4 (0.2)		4 (0.2)	0 (0)	0.2
Embarrassment	6 (0.1)	4 (0.1)	2 (0.4)	–0.3	2 (0.1)		2 (0.1)	0 (0)	0.1
Excitement	19 (0.3)	16 (0.3)	3 (0.7)	–0.4	10 (0.5)		8 (0.5)	2 (1.6)	–1.1
Fear	73 (1.3)	73 (1.4)	0 (0)	1.4	12 (0.6)		12 (0.7)	0 (0)	0.7
Gratitude	886 (15.3)	825 (15.4)	61 (13.5)	1.9	309 (16.6)		294 (17)	15 (11.7)	5.3
Joy	257 (4.4)	248 (4.6)	9 (2)	2.6	73 (3.9)		66 (3.8)	7 (5.5)	–1.7
Love	1029 (17.8)	917 (17.2)	112 (24.8)	–7.6	276 (14.9)		256 (14.8)	20 (15.6)	–0.8
Nervousness	18 (0.3)	17 (0.3)	1 (0.2)	0.1	2 (0.1)		2 (0.1)	0 (0)	0.1
Optimism	103 (1.8)	94 (1.8)	9 (2)	–0.2	168 (9)		162 (9.4)	6 (4.7)	4.7
Pride	0 (0)	0 (0)	0 (0)	0	1 (0.1)		1 (0.1)	0 (0)	0.1
Realization	64 (1.1)	61 (1.1)	3 (0.7)	0.4	17 (0.9)		16 (0.9)	1 (0.8)	0.1
Remorse	42 (0.7)	39 (0.7)	3 (0.7)	0	43 (2.3)		39 (2.3)	4 (3.1)	–0.8
Sadness	1766 (30.5)	1691 (31.7)	75 (16.6)	15.1	248 (13.4)		237 (13.7)	11 (8.6)	5.1
Surprise	26 (0.4)	22 (0.4)	4 (0.9)	–0.5	7 (0.4)		7 (0.4)	0 (0)	0.4

In the original comments category, sadness showed the largest difference between the 2 fan communities, with a 15.1% higher prevalence in BTS comments (1691/5341, 31.7%) compared to Taylor Swift comments (75/452, 16.6%), suggesting that BTS fans more frequently express their personal struggles and negative feelings when commenting on BTS content. Love and admiration showed notable differences in the opposite direction. Love was more prevalent in Taylor Swift comments with a –7.6% difference (BTS: 917/5341, 17.2%; Taylor Swift: 112/452, 24.8%), and admiration showed an –11% difference (BTS: 429/5341, 8%; Taylor Swift: 86/452, 19%).

In the “reply” category, the emotional differences were equally distinct but with different patterns. Caring showed the largest difference with an 8.5% higher prevalence in BTS replies (BTS: 242/1729, 14%; Taylor Swift: 7/128, 5.5%), followed by gratitude with a 5.3% difference (BTS: 294/1729, 17%; Taylor Swift: 15/128, 11.7%), and optimism with a 4.7% difference (BTS: 162/1729, 9.4%; Taylor Swift: 6/128, 4.7%). These differences suggest a stronger emphasis on community support and positive encouragement within BTS fan interactions. Conversely, Taylor Swift–related replies showed a higher prevalence in emotions related to fan engagement, with amusement showing a –7.8% difference (BTS: 41/1729, 2.4%; Taylor Swift: 13/128, 10.2%) and admiration showing a –6.9% difference (BTS: 150/1729, 8.7%; Taylor Swift: 20/128, 15.6%). This suggests that Taylor Swift fans engage in more lighthearted exchanges and explicit artist appreciation in their interactions.

Table 3 presents the results of multilevel logistic regression models predicting 5 key emotions in YouTube comments. After controlling for linguistic features and comment characteristics, BTS fans had significantly higher odds of expressing sadness (odds ratio [OR] 2.15, 95% CI 1.24-3.76; *P*=.007), gratitude (OR 2.18, 95% CI 1.40-3.39; *P*<.001), and caring (OR 3.10, 95% CI 1.77-5.45; *P*<.001) compared to Taylor Swift fans. These findings support our descriptive analysis and suggest that BTS fans more frequently use YouTube comment sections as spaces for emotional vulnerability and mutual support. In particular, the substantially higher odds of expressing caring emotions among BTS fans align with the strong sense of community observed in comment content. Reply comments showed distinct emotional patterns compared to original comments, with significantly lower odds of expressing sadness (OR 0.32, 95% CI 0.28-0.37; *P*<.001), love (OR 0.70, 95% CI 0.61-0.81; *P*<.001), and admiration (OR 0.74, 95% CI 0.61-0.89; *P*=.001), but dramatically higher odds of expressing caring (OR 4.38, 95% CI 3.53-5.44; *P*<.001). This pattern suggests a functional differentiation within these online communities: original comments tend to serve as venues for emotional disclosure and artist appreciation, while replies function primarily as supportive responses to these disclosures, creating a reciprocal support dynamic. First-person singular pronoun (“I”) usage showed significant associations with all 5 emotions, increasing the odds of expressing sadness (OR 1.05, 95% CI 1.04-1.05; *P*<.001) and love (OR 1.04, 95% CI 1.04-1.05; *P*<.001), while decreasing the odds of expressing gratitude (OR 0.99, 95% CI 0.98-1.00; *P*=.001), admiration (OR 0.93, 95% CI 0.92-0.94; *P*<.001), and caring (OR 0.97, 95% CI 0.96-0.98; *P*<.001). This pattern suggests that self-referential language is more commonly used when disclosing personal emotional experiences and struggles, while other, more focused emotions such as gratitude, admiration, and caring are expressed with less self-reference. First-person plural pronoun (“we”) usage had a positive association with expressing love (OR 1.06, 95% CI 1.04-1.09; *P*<.001) but a negative association with gratitude (OR 0.92, 95% CI 0.88-0.95; *P*<.001), potentially reflecting how collective identity reinforces positive emotional attachment to the artist while individual expressions dominate in gratitude.

**Table 3:** Multilevel logistic regression models predicting emotions in YouTube comments (n=30 groups for each emotion). Emotions were classified using the Bidirectional Encoder Representations from Transformers–based GoEmotions model. Random effects for video ID (n=30) were included to account for clustering within videos. Word count, length, and pronoun usage (I and we) were derived from Linguistic Inquiry and Word Count 2022 software analysis.

Variables	Sadness			Love			Gratitude			Admiration			Caring	
	OR^a^ (95% CI)	*P* value	OR (95% CI)		*P* value	OR (95% CI)		*P* value	OR (95% CI)		*P* value	OR (95% CI)		*P* value
BTS fan (reference: Taylor Swift fan)	2.15 (1.24-3.76)	.007	1.02 (0.81-1.28)		.87	2.18 (1.40-3.39)		<.001	0.98 (0.71-1.36)		.93	3.10 (1.77-5.45)		<.001
Reply comment (reference: nonreply)	0.32 (0.28-0.37)	<.001	0.70 (0.61-0.81)		<.001	0.94 (0.81-1.09)		.41	0.74 (0.61-0.89)		.001	4.38 (3.53-5.44)		<.001
Number of replies	1.00 (1.00-1.01)	.26	0.99 (0.98-1.01)		.32	1.00 (0.99-1.01)		.96	1.00 (1.00-1.01)		.25	1.00 (0.97-1.02)		.74
Length	1.00 (1.00-1.00)	<.001	1.00 (1.00-1.00)		.01	1.00 (1.00-1.00)		.006	1.00 (1.00-1.00)		.06	1.00 (1.00-1.00)		.03
Word count	1.01 (1.01-1.02)	<.001	1.00 (1.00-1.01)		.002	1.01 (1.00-1.01)		.002	1.00 (1.00-1.01)		.67	1.01 (1.00-1.01)		.003
I	1.05 (1.04-1.05)	<.001	1.04 (1.04-1.05)		<.001	0.99 (0.98-1.00)		.001	0.93 (0.92-0.94)		<.001	0.97 (0.96-0.98)		<.001
We	0.98 (0.95-1.00)	.09	1.06 (1.04-1.09)		<.001	0.92 (0.88-0.95)		<.001	0.99 (0.95-1.03)		.48	1.00 (0.98-1.03)		.77
Affiliation	1.02 (1.01-1.04)	<.001	0.99 (0.97-1.01)		.17	1.02 (1.01-1.03)		.006	0.99 (0.97-1.01)		.40	1.01 (0.99-1.03)		.37
Random effect variance	0.39 (0.18-0.82)	<.001	0.01 (0.00-0.15)		.08	0.19 (0.10-0.39)		<.001	0.05 (0.02-0.18)		<.001	0.07 (0.01-0.30)		.001

^a^OR: odds ratio.

### Emotional Expression in BTS Fan Comments

To further contextualize the quantitative emotion classification results, we examined the content of comments within each emotion category. Table 4 presents representative examples that illustrate how fans expressed mental health–related experiences and engaged in peer support within the comment sections.

Commenters across multiple emotion categories described BTS-related YouTube comment sections as spaces where they could share experiences they felt unable to disclose elsewhere. One user wrote, “Let’s be honest, the only place we can really express our feelings is in the YouTube comments, because no one knows us and won’t judge us because of that.” Another stated, “I really need to thank YouTube comment sections, because it’s the only place I can vent,” before describing experiences of childhood sexual abuse, a friend’s suicide, a parent’s cancer diagnosis, and a recent suicide attempt. Such comments suggest that the perceived anonymity and shared fan identity created conditions for disclosure that users did not find in their offline environments.

**Table 4:** Examples of emotional expression in BTS fan comments. Percentages are of BTS comments (n=5341) or BTS replies (n=1729) that the GoEmotions classifier assigned to a nonneutral emotion category. Quotations are reproduced verbatim; ellipses mark omitted text.

Emotion and type			n (%)		Examples
Sadness					
	Comment	1691 (31.7)		“My brother died of suicide last two years ago... I started having depression after when my big brother died. Two years later, I started thinking to cut myself and suicide too. But when I watch BTS ‘No More Dream’... I continue watching BTS”	
	Reply	237 (13.7)		“I feel you cause I have the same problems, and I also have no one to talk to... my heart is weaker than all the people who thinks I’m strong”	
Love					
	Comment	917 (17.2)		“I’m one of the 2 billion ARMYs that love and cherish you. Are you okay? No really are you?... as the old me was bullied for 4 years, had problems a 16-year-old should never go through”	
	Reply	256 (14.8)		“Army is a place to fall down on when you can’t stand. U r not alone. U r loved and u r part of this family”	
Gratitude					
	Comment	825 (15.4)		“I can’t go to a therapist since I’m a minor and they will tell my parents that they will be extremely angry... thank you for reading about my problems”	
	Reply	294 (17)		“Home is not the best place for me right now... People I thought cared about me, they just abandoned me... but I know I can trust ARMY”	
Admiration					
	Comment	429 (8)		“HEY YOU! Yes, you, sitting behind your screen reading this. I don’t know you and you certainly don’t know me... you’re a beautiful, wonderful, talented person”	
	Reply	150 (8.7)		“I’m proud of you for surviving 100% of your bad days. It takes a lot of courage, and that itself makes you such an amazing existence”	
Joy					
	Comment	248 (4.6)		“I had never been into a fandom that treats me like their family! I am so glad that I’ve come into this fandom, I’ve never been so attached into any bands, this is the one and only. They saved many people from depression and other mental health problems.”	
	Reply	66 (3.8)		“Ever since I became an ARMY I’ve been happier. And trying more. And not as suicidal as I was before”	
Desire					
	Comment	207 (3.9)		“I always bottled up my emotions that sometimes it blows up on the wrong time... I wanted to cry so bad, but I just can’t. It’s hurting me more. Make me start to think I was not worthy”	
	Reply	43 (2.5)		“I miss the days I used to be loved by my parents but now they hate me because I listen to BTS... I even tried committing suicide, but I was safe”	
Disappointment					
	Comment	167 (3.1)		“I have been struggling with anxiety for almost all of my life but now it’s gotten out of control... I couldn’t get into med school, keep on gaining more weight and lost all of my friends”	
	Reply	17 (1)		“I want to study hard and get good marks, but I always find myself procrastinating... I feel like I’m wasting my time unintentionally”	
Caring					
	Comment	143 (2.7)		“It’s okay to cry, you’re not weak nor sensitive. It’s alright to make mistakes, you’re not perfect. Forgive yourself of past mistakes, you weren’t as wise as today”	
	Reply	242 (14)		“I’ve seen so many comments in this comment section of ARMYs who don’t feel well and want someone to talk to... I’m not a therapist or something but I care and love every single ARMY member so if you want someone to talk to,, I’m always ready to listen.”	
Approval					
	Comment	107 (2)		“ARMY had comforted me during my panic attacks even before I liked BTS. Their music stopped me from suiciding and it is the only thing that helps me through my depression”	
	Reply	76 (4.4)		“It’s no use of going hard on yourself for that... It’s long gone into past which you can’t turn back. Pressuring and hurting yourself won’t help”	
Optimism					
	Comment	94 (1.8)		“I hope you know that the emptiness and sorrow you feel are valid... I promise you it’s only temporary and what you’re feeling is what makes you human”	
	Reply	162 (9.4)		“Dear army, I hope you will find inner peace someday. Your chest might feel heavy as if the world is against you. But you will find yourself through this”	
Fear					
	Comment	73 (1.4)		“I am here because I suffer from depression and I am afraid to tell my parents and friends... I locked myself in the room doing self-harm”	
	Reply	12 (0.7)		“I understand you. My parents also fight all the time for my brother and I, the truth at this point I’m afraid to lose him since he suffers from anxiety and even though my mom said that I wanted to take him to a psychologist my dad doesn’t understand that this is serious.”	
Remorse					
	Comment	39 (0.7)		“I have been a BTS fan since 2015, they got me through a lot over the years, but I lost touch in 2022, for a number of reasons, but now I am back at my safe place that protected me over the years from depression. I am sorry for the past year me who allowed others to treat her the way they did.”	
	Reply	39 (2.3)		“Everyone loses friends, but that’s how you know who the real ones are. Armies don’t hate you, we love you”	

Fans frequently described the BTS fan community as a surrogate family or safe space. As shown in Table 4, a joy-coded comment (248/5341, 4.6%) stated, “I had never been into a fandom that treats me like their family! I am so glad that I’ve come into this fandom... They saved many people from depression and other mental health problems.” A remorse-coded comment (39/5341, 0.7%) similarly described returning to “my safe place that protected me over the years from depression.” The framing of these spaces as nonjudgmental environments where fans could share without fear appeared across emotion categories.

Sadness was the most prevalent emotion in original comments (1691/5341, 31.7%). These comments included disclosures of depression, grief, self-harm, and suicidal ideation. As 1 commenter shared (Table 4), “My brother died of suicide last two years ago... I started having depression after when my big brother died. Two years later, I started thinking to cut myself and suicide too. But when I watch BTS ‘No More Dream’... I continue watching BTS.” Sadness-coded replies (237/1729, 13.7%) often expressed solidarity: “I feel you cause I have the same problems, and I also have no one to talk to... my heart is weaker than all the people who thinks I’m strong.”

Gratitude-coded comments (825/5341, 15.4%) and replies (294/1729, 17%) frequently linked expressions of thanks to descriptions of mental health struggles and barriers to formal care. One comment stated, “I can’t go to a therapist since I’m a minor and they will tell my parents that they will be extremely angry... thank you for reading about my problems.” A gratitude-coded reply described, “Home is not the best place for me right now... People I thought cared about me, they just abandoned me... but I know I can trust ARMY.” These examples illustrate how structural barriers (age restrictions, concerns about parental reactions, and lack of trusted relationships) shaped fans’ reliance on the fandom community.

Desire-coded comments (207/5341, 3.9%) and replies (43/1729, 2.5%) captured expressions of emotional suppression and unmet needs. One comment described, “I always bottled up my emotions that sometimes it blows up on the wrong time... I wanted to cry so bad, but I just can’t. It’s hurting me more. Make me start to think I was not worthy.” A desire-coded reply revealed, “I miss the days I used to be loved by my parents but now they hate me because I listen to BTS... I even tried committing suicide, but I was safe.”

Fear-coded comments (73/5341, 1.4%) and replies (12/1729, 0.7%) captured anxiety about disclosure and concerns about family members’ mental health. One comment stated, “I am here because I suffer from depression and I am afraid to tell my parents and friends... I locked myself in the room doing self-harm.” A fear-coded reply shared, “My parents also fight all the time for my brother and I, the truth at this point I’m afraid to lose him since he suffers from anxiety and even though my mom said that I wanted to take him to a psychologist my dad doesn’t understand that this is serious.”

Reply comments exhibited distinct emotional patterns reflecting peer support. While caring represented only 2.7% (143/5341) of original comments, it constituted 14% (242/1729) of replies. A caring-coded reply stated, “I’ve seen so many comments in this comment section of ARMYs who don’t feel well and want someone to talk to... I’m not a therapist or something but I care and love every single ARMY member so if you want someone to talk to,, I’m always ready to listen.” This explicit acknowledgment of the comment section as a space for mental health disclosure, combined with an offer of informal support, illustrates the peer support dynamics operating within these spaces.

Optimism similarly increased from 1.8% (94/5341) in original comments to 9.4% (162/1729) in replies. A comment stated, “I hope you know that the emptiness and sorrow you feel are valid... I promise you it’s only temporary and what you’re feeling is what makes you human,” while a reply offered, “Dear army, I hope you will find inner peace someday. Your chest might feel heavy as if the world is against you. But you will find yourself through this.”

Admiration appeared in both comments (429/5341, 8%) and replies (150/1729, 8.7%), often taking the form of direct encouragement. A comment addressed fellow fans: “HEY YOU! Yes, you, sitting behind your screen reading this. I don’t know you and you certainly don’t know me... you’re a beautiful, wonderful, talented person.” A reply affirmed, “I’m proud of you for surviving 100% of your bad days. It takes a lot of courage, and that itself makes you such an amazing existence.”

Love-coded comments (917/5341, 17.2%) and replies (256/1729, 14.8%) emphasized connection and belonging. One comment shared, “I’m one of the 2 billion ARMYs that love and cherish you. Are you okay? No really are you?... as the old me was bullied for 4 years, had problems a 16-year-old should never go through.” A reply emphasized community belonging: “Army is a place to fall down on when you can’t stand. U r not alone. U r loved and u r part of this family.”

Approval-coded comments (107/5341, 2%) and replies (76/1729, 4.4%) validated experiences and discouraged self-criticism. One comment described, “ARMY had comforted me during my panic attacks even before I liked BTS. Their music stopped me from suiciding and it is the only thing that helps me through my depression.” A reply counseled, “It’s no use of going hard on yourself for that... It’s long gone into past which you can’t turn back. Pressuring and hurting yourself won’t help.”

Joy-coded replies (66/1729, 3.8%) contained references to mental health improvement alongside ongoing challenges: “Ever since I became an ARMY I’ve been happier. And trying more. And not as suicidal as I was before.” Remorse-coded replies (39/1729, 2.3%) offered reassurance: “Everyone loses friends, but that’s how you know who the real ones are. Armies don’t hate you, we love you.”

## Discussion

### Principal Results

This study investigated the linguistic characteristics and emotional expressions in YouTube comments on BTS’s “sad” or “depression” playlists. Our findings reveal the diverse ways in which fans engage with the music along with other fans. For example, our findings capture how aspects of BTS music (eg, self-affirming lyrics and narratives, and melodies that carry emotion beyond language), along with the nature of fan engagement (eg, sense of community and fandom), are reflected in how fans express emotion and support one another in these spaces. Overall, our study adds to existing literature that has documented the positive effects of music engagement on mental health [19,63].

Our findings extend this work to a digital setting, YouTube comment sections. It also adds to the literature that has captured the mental health content in popular song lyrics [27,32], the impact of mental health content of popular song lyrics on important outcomes (eg, help-seeking [28]), and how fan engagement with BTS music may impact listeners’ mental health [33].

The LIWC analysis showed that BTS-related original comments were significantly longer, had a higher word count, and contained more first-person singular pronouns compared to Taylor Swift original comments. Higher use of first-person singular pronouns (“I”) among BTS fans may suggest stronger personal reflection or emotional investment; it may also reflect demographic differences, such as younger age [55] or greater psychological vulnerability. Further, the significantly higher prevalence of sadness expressed by BTS fans suggests that they are using the YouTube platform as a space to process and share their emotional struggles. This deeper emotional engagement is consistent with the music and possibly a sense of relatability to BTS’s lyrics’ message. In contrast, while expressing admiration for the artist, Taylor Swift fans appear to use the platform more for the simple enjoyment of the music.

Our attention to the individual and collective nature of music engagement fits within the Dynamic Music Engagement model [33], which sets out several routes through which music engagement can affect mental health. Specifically, the model understands music engagement in the context of the listeners, artists, fan community, and their interactions [33]. For example, we found a higher prevalence of caring, gratitude, and optimism in BTS comments compared to Taylor Swift’s comments in the “reply” category. These findings suggest the strength of the BTS ARMY community in building emotional connection as an online bonding social capital [64] and support among fans worldwide, transcending language and cultural barriers [31,65]. This finding is aligned with the fact that ARMY fandom is known as the most dedicated community among K-pop fans, providing a communal experience [63]. This also builds on prior research that found that a sense of community, reflection, connection, and comfort were key experiences shared in response to listening to BTS music [33].

Building on our NLP findings, the examination of comment content revealed that BTS music and fandom may work as a coping mechanism for fans dealing with difficult emotions, trauma, and life experiences. These findings, highlighting music’s use in managing difficult emotions and life experiences, align with prior research [21,24,66-68]. For example, fans may find comfort, emotional release, and a sense of understanding through the music and lyrics due to its capacity to address underlying emotions and experiences. This relates to seminal research [18], which delineates principles underlying music-evoked emotions, including resonance (eg, emotional contagion), understanding (eg, meaning-making), and social functions (eg, relational experiences).

We also found some evidence that music engagement served “unhealthy” functions at times. Comments classified under emotions such as sadness and desire included some evidence related to how some fans and listeners engaged with music in ways that may have intensified negative emotions or avoidant coping (eg, social isolation, blocking others out, and rumination). These findings add to some prior literature that has documented potential “unhealthy” and maladaptive functions of music, even when music is used with the intention of feeling better [24,35]. While more research is needed, our findings suggest a potential need for developing digital music interventions that maximize music’s positive impact on mental health [69] while mitigating potential risks.

Our findings also show the barriers to mental health support that BTS fans described, with many reporting a lack of access to or understanding of mental health services [70]. In the absence of formal mental health support, BTS and the ARMY community may fill the gap by providing a virtual space for understanding, validation, and encouragement [71]. It is aligned with the previous finding that ARMY forms a transcultural fandom with a strong sense of in-group identity, which is also linked to a broader identification with humanity [72]. This digital community provides a sense of belonging and mutual social support among its members and is perceived as a virtual home [73]. These findings emphasize the need for mental health resources, particularly for populations relying on online platforms for connection and communication.

### Limitations

First, the study relied on YouTube comments as the primary data source, which may not fully represent the wider BTS or Taylor Swift fandom. Fans who actively engage in YouTube comments may have distinct characteristics or experiences compared to those who do not comment. Future research could involve in-depth interviews with BTS fans from diverse backgrounds to gain a deeper understanding of their lived experiences and how BTS has impacted their mental health and well-being. This study highlights the potential of BTS music and fandom to provide mental health support and build a sense of belonging, particularly among those from Asian backgrounds, as reported in Zhang and Ong [74].

Second, the study included Taylor Swift comments as a reference group; however, the sample size for Taylor Swift comments (n=1452) was substantially smaller than that for BTS comments (n=11,772). While a comparative analysis was not the focus of this paper, future research should aim for more balanced sample sizes to ensure robust comparative analyses.

Third, the NLP tools used in this study, while powerful, have limitations in interpreting figurative and community-specific language. Fanspeak, or specialized terminology and inside jokes unique to fandoms (eg, “Easter Eggs” for Taylor Swift fans or “borahae” for BTS fans), may not be fully captured or understood by lexicon-based tools such as LIWC, which relies on fixed dictionaries that may not cover evolving slang and community-specific expressions [75], or even context-aware models such as GoEmotions, potentially leading to misinterpretation of some community-specific expressions. Further, detecting sarcasm or distinguishing between literal and figurative expressions of emotion remains a significant challenge for current NLP techniques [76].

Fourth, while the Helsinki-NLP models are well-regarded for multilingual translation [52], a formal validation of translation accuracy for every language pair, especially for subtle emotional expressions, was beyond this study’s scope. Automated translation of emotion-laden text presents inherent challenges, as affective expressions are often culturally embedded and may lack direct equivalents across languages [77,78]. This limitation extends to subsequent emotion classification: the GoEmotions model was trained exclusively on English Reddit comments [58] and culturally specific emotional expressions, such as the Korean concepts jeong (affectionate attachment) or han (deep sorrow), may be rendered into English approximations that do not fully capture the original affective meaning. Consequently, some semantic or cultural nuances in non-English comments might not have been perfectly captured, potentially affecting emotion classification accuracy for this subset of data. Our primary analyses, however, remain robust due to the large volume of English-native comments (approximately 10,129/13,224, 76.6% of the dataset) and the consistent patterns observed across languages.

Fifth, the study did not examine differences in emotional expression patterns and linguistic characteristics across demographic groups within the BTS fandom, such as age, gender, or geographic location. Public YouTube comment data does not include user demographic information, and the YouTube application programming interface does not provide access to commenter demographics [11,79]. This limitation is common across social media research, where demographic variables must often be inferred rather than directly observed. Future research could investigate how these factors shape fans’ engagement with music and its impact on their mental health.

Sixth, our cross-sectional design does not capture temporal trends in emotional expression across the 2018-2024 study period. Future research could build on these findings by using longitudinal designs to examine how emotional expression patterns within fan communities evolve over time. The study period (2018-2024) covers events that may shape fan engagement in ways that warrant temporal analysis, including the COVID-19 pandemic, which affected both online mental health discourse [80] and music listening behaviors [81], and artist-specific events such as album releases and mental health advocacy campaigns.

Finally, while NLP provides insights into large-scale linguistic patterns, it is not designed to measure the depth of human experience or to work as a diagnostic instrument for mental health conditions [82,83]. Our findings reflect patterns of emotional expression and supportive interaction as articulated in text, rather than directly assessing internal psychological states or predicting clinical outcomes. The study identifies expressions of peer support and self-expression, but should not be equated with clinical assessment. Future research, particularly with qualitative methods, can provide a deeper context.

Overgeneralizing from commenters to the fandom as a whole also risks stereotyping fan communities [84].

Despite these limitations, this study provides a foundation for future research on the role of music and fandom in mental health, particularly across diverse cultural contexts. Addressing these limitations and ethical considerations can enhance the validity and impact of future studies, contributing to a deeper understanding of how music and artist fandoms can support mental health. Methodologically, the NLP methods demonstrated in this study offer accessible tools for mental health researchers and practitioners interested in understanding emotional expression in online communities. LIWC and pretrained emotion classification models such as GoEmotions provide validated approaches that do not require model development, making large-scale analysis of fan discourse feasible for interdisciplinary teams working at the intersection of mental health and digital media.

### Conclusions

This study has several implications related to mental health. Our findings provide a resource to clinicians who wish to integrate BTS music and its themes into their work, particularly with clients from Asian and Hispanic/Latino backgrounds. Music that matches fans’ emotions and experiences can be a useful component of psychosocial interventions to enhance engagement and therapeutic benefits, especially for young people [23]. The language distribution in the methods section, which revealed a significant proportion of Spanish (719/11,772, 6.11%) and Portuguese (222/11,772, 1.89%) comments among BTS fans, highlights the relevance of BTS music to Hispanic/Latino populations.

The supportive nature of the BTS ARMY community emphasizes the importance of promoting peer support and community building for individuals struggling with mental health issues. Mental health organizations can build similar online communities and safe spaces for people from diverse cultural backgrounds to connect and share their experiences, such as the example that BTS ARMY Help Center partnered with Crisis Text Line for mental health support [85].

The challenges fans face in accessing mental health support point to the need for culturally sensitive and accessible services, with mental health providers striving to create inclusive environments and tailor their approaches to the unique needs and preferences of people from diverse cultural backgrounds. The role of BTS music on fans’ mental health and well-being demonstrates the potential for music and celebrity influence to promote positive mental health messages and reduce stigma [86,87]. Collaborations between mental health organizations and influential artists such as BTS can help raise awareness and encourage help-seeking behaviors in ways that draw on digital technology [88,89]. This does not imply using platforms such as YouTube directly as therapeutic tools, as they are not designed for such purposes and lack the necessary safety and privacy infrastructures for clinical intervention. Instead, collaborations could involve (1) artists partnering with established mental health advocacy groups (eg, National Alliance on Mental Illness and Active Minds) or global health organizations (eg, World Health Organization and United Nations Children’s Fund [UNICEF]) to cocreate awareness campaigns, as exemplified by BTS’s LOVE MYSELF campaign with UNICEF [90]; (2) insights from online fan interactions informing the design of bespoke, ethically sound digital mental health tools; and (3) artists using their platforms to direct fans toward verified mental health resources and evidence-based interventions [86,87].

Our findings suggest several potential applications for mental health practitioners. Clinicians working with those from diverse cultural backgrounds might ask about clients’ engagement with music fandoms as an entry point for discussing emotional experiences and coping strategies. The prevalence of comments expressing music as a coping mechanism and community belonging suggests that fandom participation may act as an informal supplement to formal mental health care for some individuals. However, clinicians should also remain alert to potentially maladaptive patterns [25,34,35], such as excessive reliance on parasocial relationships or using music to avoid difficult emotions. Digital mental health interventions might consider drawing on the positive aspects of music engagement and community support while providing guidance toward additional evidence-based coping strategies. Such interventions could also benefit from understanding how fan communities operate across multiple platforms. Fan interactions on YouTube, with its extended comment format, may differ from those on Twitter, Reddit, Weverse, or TikTok, each with distinct affordances and community norms [91,92]. Mental health practitioners and digital intervention designers seeking to engage with fandom communities should consider these platform-specific dynamics when developing outreach strategies.

## Abbreviations

ARMY: Adorable Representative MC for Youth
BERT: Bidirectional Encoder Representations from Transformers
LIWC: Linguistic Inquiry and Word Count 2022 software
NLP: natural language processing
OR: odds ratio
UNICEF: United Nations Children’s Fund
